# Pharmacodynamic material basis of traditional Chinese medicine based on biomacromolecules: a review

**DOI:** 10.1186/s13007-020-00571-y

**Published:** 2020-02-28

**Authors:** Wen-Jin Zhang, Sheng Wang, Chuan-zhi Kang, Chao-geng Lv, Li Zhou, Lu-Qi Huang, Lan-Ping Guo

**Affiliations:** 1 State Key Laboratory of Dao-di Herbs Breeding Base, Joint Laboratory of Infinitus (China) Herbs Quality Research, National Resource Center for Chinese Materia Medica, China Academy of Chinese Medical Sciences, Beijing, 100700 China; 2 College of Pharmacy, Ningxia Medical University, Yinchuan, 750004 China

**Keywords:** Traditional Chinese medicines, Pharmacological activities, Mechanisms, Biomacromolecules

## Abstract

Biomacromolecules, the first components of bioactive substances in traditional Chinese medicines (TCM) have wide bioactivity-related efficacy but have not yet been fully appreciated compared to small molecule components. The present review brings a novel and systemic point of view to deepen the understanding of the pharmacodynamic material basis of TCM based on biomacromolecules (polysaccharides, proteins and nucleic acids). Biomacromolecules have been, are and will have considerable roles in the efficacy of Chinese medicine, as evidenced by the number of biological activities related to traditional clinical efficacy. The direct and indirect mechanisms of biomacromolecules are further accounted for in a variety of neurotransmitters, hormones, and immune substances to maintain immune function in both sensitive and stable equilibrium. The biological functions of biomacromolecules have been elaborated on in regard to their roles in the process of plant growth and development to the relationship between primary metabolism and secondary metabolism and to the indispensable role of polysaccharides, proteins, and nucleic acids in the quality formation of TCM. Understanding the functional properties and mechanisms of biological macromolecules will help to demystify the drug properties and health benefits of TCM.

## Highlights

- Biomacromolecules play considerable roles in Chinese medicine, as their biological activities are related to TCM efficacy and their biological activities are closely correlated to their chemicophysical properties.
- Oligosaccharides, oligopeptides and miRNA can be directly absorbed and play direct and indirect roles in efficacy with multichannel, multilevel, multitarget processes.
- This review also describes the shortcomings of related research and presents some suggestions for reference.

## Background

Traditional Chinese medicine (TCM) has been used for disease prevention and treatment throughout the ages and is thought to have profound impacts on human survival and reproduction [1]. Compared to modern medicine, pharmacotherapy using natural substances can be currently regarded as a very promising future alternative to conventional therapy [2]. During long-term clinical practice, TCM is mostly prepared by boiling with water to generate decoctions (water extracts) for oral administration [3]. However, the scientific connotations and mysteries of TCM remain largely unknown. Completely differing from Western medicines, the chemical compositions of which are simple and definite, TCM decoctions house complex matrixes and undefined active components. Which components contribute to therapeutic effects and how they synergistically work remain unknown. To innovate TCM, illumination of these issues is necessary [4]. The chemical diversity of TCM has been well-defined by accumulated phytochemical studies. Small molecules (generally MW < 1 kDa) are the most representative [5]. Intensive multidisciplinary research studies have provided abundant information of small molecules in TCM by elucidating chemical structures, evaluating pharmacological activities, determining systemic exposure, as well as exploring action targets [6, 7]. It has been adequately demonstrated that glycosides, such as flavone glycosides, saponin and iridoid glycosides, which are normally polar chemicals and occur frequently in TCM decoctions [8], are often metabolized to secondary glycosides and/or aglycones with better bioavailability and bioactivity by enzymes encoded in intestinal bacteria [9]. By sufficient elaborations, small molecules are commonly deemed to be the crucial bioactive chemicals that are responsible for the curative effects of TCM. In contrast, the role of TCM macromolecules is not yet clear, as they are generally indigestible by oral administration and hardly absorbable in the gastrointestinal tract [10]. Clouded by scientific cognition, the biomacromolecules of TCM are currently often under-appreciated or even disregarded. For instance, polysaccharides, proteins and nucleic acids are normally removed as impurities to meet the requirements of purity and dosage amounts of the final TCM preparation in modern industrialized production [11]. Even then, scientific research on TCM decoctions often excludes them from biologically key chemicals [12]. Obviously, such situations not only deviate from the traditional usage of TCM but also lack scientific evidence.

In the last three decades, numerous biological activities of polysaccharides, proteins, polysaccharide-protein complexes and nucleic acids (mainly miRNAs) have been identified from TCM and have even been used as sources of therapeutic agents [2, 13]. Polysaccharide is a kind of natural macromolecular polymer that is usually composed of more than 10 monosaccharides through glycosidic linkages in linear or branched chains, with molecular weights of tens of thousands or even millions [14]. Polysaccharides are widely existing in plants, microorganisms, algae, and animals. Similar to proteins and polynucleotides, polysaccharides are essential macromolecules in life activities and play important roles in cell–cell communication, cell adhesion, and molecular recognition in the immune system [15]. In recent years, polysaccharides isolated from natural resources, plants, animals, fungi, seaweed have attracted increasing attention because of their wide variety of pharmacological activities, such as antitumor, immunomodulation, anti-oxidation, and anti-inflammatory effects, etc. [16]. For instance, astragalus polysaccharides, ginseng polysaccharides, lentinan, fucoidan, pachman, and Coriolus versicolor polysaccharides are already polysaccharide drugs in domestic and foreign markets [2]. According to the traditional theory of protein absorption, proteins can only be absorbed by the body after being decomposed into free amino acids in the body. Until the study of Newey and Smith in 1960, it was found that the digestive products of proteins in the small intestine consisted of not only amino acids but also a large number of oligomers [17]. In fact, the study showed that most of the digestive end products of proteins in the digestive tract were oligomers. Moreover, oligopeptides can completely enter systemic circulation through intestinal mucosa cells. Compared with other bioactive substances, bioactive peptides exert great influence in metabolic regulation, even at small dosages [18]. Various kinds of bioactive peptides, which possess similar functions related to their traditional known efficacy, have been identified from TCM; thus, the real efficacy in some TCM, especially protein-rich TCM, may be derived from their bioactive peptides. miRNAs are a class of 19- to 24-nucleotide-long non-coding RNAs that act as post-transcriptional regulators of gene expression in eukaryotes [19]. A recent study reported an unexpected finding that plant miRNAs that are ingested from plant sources can pass through the gastrointestinal tract, enter the blood, accumulate in tissues and regulate endogenous gene expression [20] or are translated to express bioactive polypeptides or proteins, or regulate the function of corresponding cells; the expression products can also be transported to the corresponding target organs through the blood circulation to perform their special biological functions.

In addition to pharmacological activities, polysaccharides, proteins and other biological macromolecules play important physiological functions in medicinal plants. Increasing amounts of evidence have shown that carbohydrates are involved in reproductive development, growth, stress and other processes and are the determinant of molecular recognition in many physiological and pathological processes. Proteins are the specific executor of life activities. In growth, development and various physiological and pathological processes, although the genome is usually stable, the composition of the expressed proteome is continually changing. Approximately 90% of cell wall components are polysaccharides. As the first defense layer of plants, the cell wall plays an indispensable role in response to biotic and abiotic stresses. More than 1/3 of the proteins in organisms are glycoproteins, which are widely distributed in the cell wall, cell membrane and cytoplasm, including many enzymes, lectins, structural proteins and other protein types [21]. Glycoproteins are often the first receptors to receive external signals and are responsible for the information exchange between cells and the outside world [22]. Previous studies have shown that miRNAs play a widespread and unimaginable role in life activities by inhibiting target mRNA translation and reducing corresponding protein synthesis, with far-reaching and complex effects on physiological and biochemical activities, such as growth and development, organ formation, gene expression, gene recombination, cell cycle, stress and disease occurrence and development process. Other studies have also documented the importance of miRNAs that are transmitted from one species to another and facilitate cross-talk and interspecies communication [23]. Thus, scientists have begun to explore the function of biomacromolecules as the awareness of their necessary role in the growth and quality formation of medical plants has increased. It is undeniable that the small molecule secondary metabolites are the important material basis of TCM. However, from the perspective of the biosynthesis pathway, secondary metabolism is connected with primary metabolism from several main branches; furthermore, some key products of primary metabolism are the initiators of secondary metabolism [24].

The function of biomacromolecules in TCM has been evidenced as above. However, the missing related knowledge has clouded our minds to better understanding their role in the quality formation of TCM. (1) Most of the characterizations of TCM macromolecules’ biological activities are completely separated from the efficacy of the TCM itself. Up to now, there has been no review on the bioactivities of polysaccharides, proteins or miRNAs related to the efficacy of TCM. (2) Current research has been dwarfed by the indeterminacy and unsystematic biological function of macromolecular substances in the growth and quality formation of medicinal plants. This review will open a window and present an idea.

### Biological activities of biomacromolecules based on its efficacy

TCM is a practical experience medicine. Clinical efficacy is the first measure used to evaluate the quality of TCM. The key to biomacromolecules in the medicinal substance basis of TCM is the research of pharmacological activities and clinical applications associated with the efficacy. Recently, accumulated evidence has demonstrated that polysaccharides, proteins (including peptides) and nucleic acids (mainly miRNAs) [25, 26] have a broad spectrum of biological effects, especially immunomodulation, anti-tumor and antioxidant activities [2]. Owing to their safe and nontoxic properties, some bioactive polysaccharides, proteins or miRNAs have been widely used in biochemical and medical practical applications [15]. However, the current findings are just a tip of the iceberg of Chinese medicine activities. It can be easily found that the vast majority of TCM have certain immunoregulation, antioxidant and even anti-tumor activities, and the material basis is mainly biomacromolecules. The unique roles of such biomacromolecules have been neglected in the study of the pharmacodynamic material basis of Chinese medicine. Moreover, the effects of macromolecular matter are closely related to their structural features, defined by molecular weight, composition and sequence. The following provides a perspective (Table 1).

**Table 1:** Biological activities of biomacromolecules based on its traditional efficacy

Resources	Polysaccharides/Proteins/miRNA	Molecular weight (KDa)	Compositions/structure	Bioactivities	Effects/mechanisms	Refs.
*Panax Ginseng*	Glycopeptides (GGP)	6	Rhamnose, arabinose, galactose, glucose (0.46:1.61:1:2.37); contained 1.6–27% polypeptides (consisted of 16 amino acids, Asp, Glu, Pro, and others)	Anti-hyperglycemia	GGP induced the pronounced decreases in blood glucose and liver glycogen levels in both normal and hyperglycemic animals	[30]
	Glycopeptides (GMP)	12.38	Arabinose (21.9%), galactose (22.6%), glucose (14.8%), rhamnose (5.8%), uronic acid (32.7%), and protein (2.2%)	Immunoregulatory	GMP increased the lysosomal phosphatase activity and the phagocytic index of peritoneal macrophages and its immunostimulating effects may be due to its ability to stimulate the production of reactive oxygen intermediates	
	Oligopeptides (GOP)	< 1	Unknown		GOP enhanced innate and adaptive immune responses in mice by improving cell mediated and humoral immunity, macrophage phagocytosis capacity and NK cell activity	[32]
	Polysaccharides (PGP)	Unknown	Unknown	Qi-invigorating and anti-fatigue	PGP inhibited mitochondrial injury and swelling and improved energy metabolism in a concentration dependent manner	[29]
		Unknown	α (1 → 6) Glucopyranoside and β (2 → 6) fructofuranoside (5:2)	Immunoregulatory	PGP enhanced the humoral immune response to orally delivered antigen, mediated by CCL3 via cyclooxygenase	[28]
	Polysaccharides (Two neutral and six acidic fractions)	3.5–110	Starch-like polysaccharides, pectic arabinogalactans and RG-I-rich and HG-rich pectins		Both the neutral and acidic polysaccharides were potent B and T cell stimulators	[31]
*Pseudostellaria heterophylla*	Polysaccharides (PRP)	Unknown	Unknown	Anti-fatigue	PRP is beneficial to chronic fatigue syndrome, and the underlying mechanisms of action involve neuroendocrine and immune systems	[34]
*Glehniae radix*	Polysaccharides (GRP)	13.3	α‑D‑Glucan containing (1 → 6)-linked and (1 → 3)-linked backbone with a branch of one (1 → 6)-linked and terminal glucoses submitting at the C-4 position every fourteen residues	Immunoregulation, anti-inflammatory and anti-tumor	GRP exhibited inhibition against A549 cells proliferation and NO production in RAW264.7 cells, and displayed promotion for proliferation of mouse spleen lymphocytes and RAW264.7 cells	[36]
*Astragalus membranaceus*	Polysaccharides (APS)	6–20	α-1,4 (1,6) Glucan, arabinose galactose polysaccharides, rhamnose galacturonic acid polysaccharides, and arabinose galactose protein polysaccharide	Immunoregulatory	APS suppressed CD4^+^ CD25^+^Treg activity, at least in part, via binding TLR4 on Tregs and triggered a shift of Th2 to Th1 with activation of CD4^+^ T cells in burned mice	[38]
		69	Unknown	Immunoregulatory	APS increased the level of cytokines including TNF-α, GM-CSF and the production of NO. NF-κB protein levels are increased in response to APS. Blocking NF-κB with specific inhibitor resulted in decreased levels of NO and TNF-α	[35]
		20.7	α-d-Glucan residues, APS has repeating (1 → 4)-linked backbone with a (1 → 6)-linked branch every 10 residues	Immunoregulatory	APS was able to stimulate activity of purified mouse B cells without promoting T cell proliferation	[37]
		11	Rhamnose, glucan, galactose, arabinose (1.19:72.01:5.85:20.95)	Anti-inflammation	APS reduced cell accumulation, swelling and arthritic index of the joints and serum concentrations of TNF-α and IL1-β in a dose-dependent manner in AA rats. Synovial cell apoptosis was elevated by APS and accompanied by increased Bax protein and decreased Bcl-2 protein	[39]
		36,300	APS:APS I, II, and III (1.47:1.21:1). APS I consisted of d-glucose, d-galactose, l-arabinose (1.75:1.63:1). Both APS II and APS III are dextrans, the linkage mode o is mainly α-(1 → 4) linkage, and in which α-(1 → 6) linkages are exiguous	Anti-atherosclerosis and anti-diabetes	APS regulated part of the insulin signaling in insulin resistant skeletal muscle, and that APS could be a potential insulin sensitizer for the treatment of type 2 diabetes	[38]
*Rhizoma dioscoreae*	Polysaccharides (YP-1)	42	Glucose, mannose, galactose (1:0.37:0.11); (1 → 3)-α-glucopyranose as a main chain and -β-galactopyranose-[(1 → 2)-α-mannopyranose]3-(1 → 2)-α-mannopyranose-(1 → 6)-as a side chain	Immunoregulatory	YP-1 stimulated ConA-induced T lymphocyte proliferation and its branches are extremely important for the expression of the enhancement of the immunological activity	[41]
	Glycoproteins	Unknown	Unknown	Immunoregulatory	*Rhizoma dioscoreae* glycoproteins promoted immunity by affecting thymus organ and phagocytic index of mice	[124]
	Polysaccharides (DOTP-80)	123	Glucose, galactose, mannose, arabinose (23.7:9.3:17.8:1.0)	Hypoglycemia	DOTP-80 had strong hypoglycemic activity. DOTP-80 increased SOD activity in alloxan induced diabetic mice and increased glucose disposal in diabetic rats	[42]
*Ganoderma lucidum*	Polysaccharides (Gl-PS)	8.849	Gl-PS consisted of d-glucose with minor amounts of galactose, arabinose and mannose (22.4:1.9:1.0:2.1)	Anti-hyperglycemia	Gl-PS decreased fasting plasma glucose, TC and TG in STZ-induced diabetes rats due to its antioxidant activities and ability to inhibit NO production caused by STZ	[44]
	Proteins (Lz 8)	12.722	Lz 8 consisted of 110 amino acid residues with an acetylated amino terminus	Immunoregulatory	Lz 8 induced phagocytosis of macrophages	[43]
	Proteins (GCL)	18	Its N-terminal sequence displays slight similarity to a lectin from fungal immunomodulatory proteins		Unknown	[110]
*Coriolus versicolo*	Polypeptides (PSK); polysaccharide peptides (PSP)	Unknown	PSK consisted of a polypeptide moiety to which polysaccharide β-d-glucan chains are attached; approximately 62% is polysaccharide and 38% is protein. PSP was a closely related protein-bound polysaccharide	Immunoregulatory	PSP and PSK enhanced immunoregulation by inducing production of IL-6, interferons, immunoglobulin-G, macrophages, and T-lymphocytes	[46]
	Polysaccharide peptides (PSP)	100	PSP is composed of 74.6% glucose, 4.8% xylose, 2.7% galactose, 2.4% fructose and 1.5% mannose. 18 amino acid were contained	Anti-virus	PSP induced the production of serum interferon and in vitro studies suggested that PSP may be useful against HIV-1 infection	[45]
Licorice	Arabinogalactan proteins	Unknown	contained 65% polysaccharides on the basis of fraction (52% arabinose and 22% galactose were the major neutral sugars together with 6% rhamnose and 2% fucose)	Antitussive	Licorice polysaccharides showed the ability to reduce citric acid-induced cough in awake guinea pigs after oral administration	[49]
	miRNA	Unknown	Unknown	Immunoregulatory	miRNA inhibited the differentiation of T cells and the expression of genes related to inflammation and apoptosis	[48]
*Schisandra Chinensis*	Glycopeptides (SCP)	265	Mannose, glucose, galactose and arabinose (1.32%, 54.41%, 44.10%, 0.17%); glycoprotein, the protein part of SCP consisted of 12 amino acids, Total protein content of SCP was 2.30%	Anti-fatigue	SCP had therapeutic effect on chronic fatigue syndrome was partially related to TCA cycle metabolic pathways and the alanine, aspartate and glutamate metabolism	[51]
	Polysaccharides (SCP-IIa)	7.7	Homogeneous polysaccharide without protein and nucleic acid	Immunomodulatory	SCP-IIa increased the thymus and spleen indices, as well as the pinocytic activity of the peritoneal macrophages in immunosuppressed mice	[125]
	Polysaccharides (SCFP-1)	31.8	Glucose, arabinose, Rib, rhamnose, xylose, galactose, mannose (302.2:133.6:11.9:2.7:1.7:1.4:1), contained 96.9% carbohydrate and 14.2% uronic acid	Antitussive	SCFP-1 showed remarkable suppressive effects on cough and attenuated inflammatory cells in BALF and some typical characteristics of nonspecific airway inflammation in animals exposed to CS	
	Polysaccharides (SCP)	Unknown	Unknown	anti-AD	SCP improved the cognition of mice, and it played an anti-AD role by activating the NF-κB/MAPK pathway to alleviate neuroinflammation	[50]
*Angelica sinensis*	Polysaccharides (AAP)	52	Mannose, rhamnose, galacturonic acid, glucose, galactose and arabinose (0.44:1.00:10.52:7.52:8.19:14.43), where the molar percentage amount of galacturonic acid was 25.0%	Immunomodulatory	AAP improved the mRNA expression of toll-like receptor 4, and the pretreatment of macrophages with anti-TLR4 antibody significantly blocked AAP-induced NO release, TNF-α secretion, and the increase of iNOS activity	[126]
	polysaccharide (ASP)	Unknown	Unknown	Hemopoiesis	ASP inhibited the expression of signal transducer and activator of transcription 3/5 and mothers against decapentaplegic proteins 4 in liver and stimulated the secretion of erythropoietin. and is likely to involve the PI3K/AKT pathway	[127]
	Polysaccharides (ASPS)	8 and 76	The raw polysaccharides (ASPS) contained ASP I and II (7.41:1); APS I and II consisted of arabinose, galactose, and glucose		The hematopoietic activity was improved by stimulation of IL-6 and GM colony-stimulating factor secretion	[53]
	Proteins	17–90	Unknown	Righting and dispeling evil	*Angelica* decoction proteins can scavenge DPPH free radicals, have a very significant proliferation effect on normal human liver cell line L-02, and an inhibitory effect on human leukemia cell line K562	[54]
*Radix Rehmanniae* Preparata	Polysaccharides (RRPP)	Unknown	Rehmannan SA and rehmannan SB in RRPP. They were commonly composed of l-arabinose:d-galactose:l-rhamnose:d-galacturonic acid (10:10:1:1) (rehmannan SA) and (14:7:3:8) (rehmannan SB)	Anti-fatigue	RRPP increased the storage of hepatic glycogen and the decrease of the accumulation of SUN and BLA	[55]
*Maidong*	*Liriope spicata* polysaccharide (LSP), *Ophiopogon japonicus* polysaccharide (OJP), *Liriope muscari* polysaccharide (LMP)	4.742, 4.925 and 4.138	Fruf-(2 → , 2 → 2)-Fruf-(6 → , → 6)-Glcp-(1 → and → 1,2)-Fruf-(6 → with a molar ratio of 5.0:18.2:1.0:5.3 (LSP), 6.8:15.8:1.0:5.8 (OJP), 8.3:12.3:1.0:3.9 (LMP)	Anti-diabetes	LSP, LMP and OJP increased the expression of PI3K, AKT, InsR, PPARγ and decreased the expression of PTP1B in mRNA level and protein level in IR HepG2 cells. Furthermore, glucose consumption was increased after treated with polysaccharides	[58]
*Lycium barbarum*	Polysaccharides (LBP)	150 (LBPF1-4)、293 (LBPF5)	LBP contains LBPF1, LBPF2, LBPF3, LBPF4 and LBPF5	Immunomodulatory	LBP and LBPF1-5 activated transcription factors NF-κB and AP-1 by RAW264.7 macrophage cells, induced TNF-α, IL-1β, IL-12p40 mRNA expression, and enhanced TNF-α production in a dose-dependent manner	[62]
	Polysaccharides (LbGp4)	214.8; carbohydrate content 85.6	Arabinose, galactose, rhamnose, glucose (1.5:2.5:0.43:0.23)	Immunomodulatory	Immunostimulatory effect by activating the expression of NF-jB and activator protein 1 (AP-1)	[61]
	Polysaccharides (LBP3p)	157	Galactose, glucose, rhamnose, arabinose, mannose, xylose (1:2.12:1.25:1.10:1.95:1.76)	Immunomodulatory	LBP3p induced immune responses by increasing the expression of IL-2 and TNF-α at both mRNA and protein levels; inhibiting the growth of transplantable sarcoma while increasing macrophage phagocytosis, spleen lymphocyte proliferation and CTL activity	[128]
	Glycocojugates LBP-X	23.7 to 214.8	Rhamnose, galactose, glucose, arabinose, mannose, and xylose (4.22:2.43:1.38:1.00:0.95:0.38). LBP-X contained 17 amino acids (8.46%)	Anti-diabetes	Crude LBP and purified polysaccharides fraction reduced the blood glucose levels and serum TC and TG concentrations while increased HDL levels	[59]
	Polysaccharides (LBP)	Unknown	Unknown	Enhancing physical strength and ameliorating physical fatigue	LBP prolonged the weight-loaded swimming time, increased the content of hepatic glycogen and prevented the increase of blood lactic acid of mice after swimming	[129]
	Polysaccharides (LBP)	Unknown	Unknown	Ameliorating male infertility	LBP attenuated diabetic testicular dysfunction via inhibition of the PI3K/Akt pathway-mediated abnormal autophagy in male mice	[63]
*Alpiniae oxyphyllae*	Polysaccharides (PAOF)	287	The contents of carbohydrate, protein, sulfated group and uronic acid from PAOF were 95.25%, 4.28%, 6.12% and 3.13%	Anti-urinary incontinence	PAOF reduced the urination volume, Na^+^, Cl^−^ emission and increase K^+^ excretion of hydruric model rats. And increased the content of aldosterone and antidiuretic hormone. The coefficients of spleen, thymus and adrenal were improved by PAOF	[64]
*Cordyceps sinensis*	Polysaccharides (CPS-2)	43.9	α-(1 → 4)-d-glucose and α-(1 → 3)-d-mannose, branched with α-(1 → 4,6)-d-glucose every twelve residues on average	Protection of chronic renal failure	CPS-2 relieved renal failure caused by fulgerizing kidney	[71]
	Polysaccharides (UM01-S4)	22.559	α-(1 → 2)-Manp core. The side chains were composed of β-(1 → 2)-Galf, β-(1 → 4)-Glcp, α-Galp A, and α-Manp units, which attached to the mannan core at the O-6 position	Immunomodulatory	UM01-S4 exhibited macrophages proliferation, phagocytosis, and release of NO and cytokines. The mechanism of macrophage regulation related to the activation of the MAPK and NF-κB signalling pathways	[65]
*Pilose antler*	Proteins (PCP)	35.6	PCP was a disulfide-linked heterodimeric glycoprotein subunits with *N*- and *O*-glycosylation	Immunoregulatory	*Pilose antler* proteinase stimulated the proliferation of mouse spleen cells and inhibited the proliferation of T lymphocytes induced by Con A	[73]
	Polypeptides	Unknown	The peptide consisted of 34 amino acids	Improving sexual	*Pilose antler* polypeptide increased the content of LH and T in the plasma of male rats and reduce the content of PRL in the plasma of female rats	[72]
	Polypeptides (PSAB)	10–70	PSAB is a mixture of 5 proteins, which contains 17 amino acids	Anti-fatigue	PSAB enhanced the anti-fatigue effect and adrenal function of the body, protected the paint stressed mice, and increased the number of red blood cells and the content of hemoglobin	[75]
*Semen cuscutae*	Polysaccharides (C-7WR1, C-7WR2 and C-7WR3)	75.9; 32.3 and 22.5	Fructose, mannose (0.02:1) (C-7WR1); fructose, mannose, xylose, arabinose (0.01:1:0.14:0.33) (C-7WR2); fructose, mannose, xylose, arabinose (0.01:1:0.10:0.47) (C-7WR3). They mainly contained mannose and had no nucleic acid and protein	Nourishing kidney	*S. cuscutae* polysaccharides nourished kidney-yang by increasing the levels of testosterone and estradiol, decreasing the level of blood urea nitrogen, improving immune function, possessing antioxidant effect	[76]
*Prunellae Spica*	Polysaccharides (PSP-2B)	32	The major sugars of PSP-2B were arabinose, galactose and mannose, glucose and uronic acids. PSP-2B also contained 2.98% protein	Anti-virus	PSP-2B exhibits activity against herpes simplex virus (HSV)	[83]
*Lonicera japonica*	miR2911	Unknown	Unknown	Anti-virus	miR2911 in *Lonicera japonica* decoction can be fed into mice by gavage and play a direct role in influenza virus	[78]
*Trichosanthis radix*	Proteins (TCS)	24	TCS was a single-chain protein with 247 amino acid residues including a 23-amino acid N-terminal signal peptide and a 19-amino acid C-terminal pro-peptide	Anti-virus	TCS inhibited replication of human immunodeficiency virus type 1	[111]
		Unknown	Unknown	Selective immunoregulatory	Non-cytotoxic concentrations of TCS suppress the activation, multiplication and differentiation of T and B cells but do not suppress the activation of natural killer cells	[82]
*Anemarrhena asphodeloides*	Polysaccharides (AABP)	1110	d-Mannose, l-rhamnose, d-galacturonic acid, d-glucose, d-galactose and l-arabinose (1:0.04:0.53:0.11:0.33:0.25)	anti-constipation	AABP could treat constipation by regulating the gastrointestinal hormones and neurotransmitters to improve intestinal motility and water metabolism	[84]
*Rehmannia glutinosa*	Polysaccharides (RGP)	63.5	Rhamnose, arabinose, mannose, glucose and galactose (1.00:1.26:0.73:16.45:30.40)	Anti-diabetes	RGP meliorated hyperglycemia, hyperlipemia, vascular inflammation and oxidative stress in STZ-induced diabetic mice	[86]
		Unknown	Unknown	Immunoregulatory	RGP stimulated lymphocyte proliferation and the growth rate of T cell and IL-2 and IFN-γ production of T lymphocyte were significantly upregulated	[87]
*Senna obtusifolia*	Proteins	19.7	Its secondary structure has 12.5% α-helix, 55.6% β-sheet, and 31.9% random coil	Cholesterol lowering	Cholesterol-lowering protein inhibited cholesterol biosynthesis in Chinese hamster oocytes	[88]
Earthworm	EFE-III-1	700	Unknown	Thrombolysis	I-labeled fibrinogen showed that EFEs by oral administration had a significant fibrinolytic effect on clots in blood vessels	[92]
*Salviae miltiorrhizae*	Polysaccharides (SMP1)	550	Galactose, glucose, fucose, rhamnose, arabinose and mannose (1.0:1.2:0.3:1.5:1.3:1.9). SMP1 contained 91.3% of total carbohydrate, 2.81% of uronic acid and 4.34% of protein	Protection of cardiomyocytes	SMP prevented myocardial infarction induced by I/R by improving oxidative stress and inhibiting myocardial cell apoptosis	[93, 94]
*Achyranthes bidentata*	Polysaccharides (ABPB-3)	77.23	→ 4)-α-d-GalpA-(1 → , → 2,4)-α-l-Rhap-(1 → , → 5)-α-l-Araf-(1 → , → 2,3,5)-α -l-Araf-(1 → , → 3)-β-d-Galp-(1 → , → 3,4,6)-β-d-Galp-(1 → , terminated with α-l-Araf, α-LRhap and β-d-Galp	Anti-osteoporosis	ABP increased the bone mineral density, bone mineral content, trabecular thickness, trabecular number and biomechanical properties of ovariectomized (OVX) rats	[96]
	Polysaccharides (ABW70-1)		(2 → 1)-linked-β-d-fructofuranosyl (Fruf), (2 → 6)-linked-β-d-Fruf and (2 → 1,6)-linked-β-d-Fruf residues, and terminated with fructose and glucose residue		ABW70-1 stimulated the osteogenic differentiation of MC3T3-E1 cells by promoting cell proliferation, ALP activity, mineral nodules formation and the gene expression of Osx, Ocn and Bsp.	[95]
	Polysaccharides (ABP)	Unknown	Unknown	Anti-physical fatigue	ABP had clear anti-physical fatigue effects which extended the exhaustive swimming time of the mice, increased the liver glycogen and muscle glycogen contents and decreased the blood lactic acid and blood urea nitrogen contents	[20]
*Gastrodia elata*	Polysaccharides (PGEB-3H)	28.8	Glucose; α-1,4-glucan and α-1,4,6-glucan	Neuroprotection	PGEB-3H improved the learning and memory ability of mice with scopolamine-induced memory disorders by increasing the Ach content in brain tissue	[98]
	Polysaccharides (PGE)	1540	Glucose; α-1,4-glucan, α-1,3-glucan andα-1,4,6-glucan		PGE was high-molecular-weight polysaccharide which exhibited Angiotensin-I converting enzyme (ACE) inhibitory activity. Its inhibition rate on ACE was calculated as 74.40% and the IC50 value was 0.66 mg/mL	[97]

Tonic Chinese medicine includes tonifying Yin, Yang, blood and Qi. Tonic Chinese medicine is used to deficiencies of the body and achieves the purpose of strengthening the body. Modern research has shown that the tonic effect of TCM is mainly the immune regulation of the body; thus, research on the immune polysaccharides and proteins of tonic Chinese medicine has been more in-depth. The use of *Panax ginseng* (*P. ginseng*) in TCM dates back to approximately 5000 years ago thanks to its several beneficial and healing properties [27]. Over the past few years, extensive amounts of preclinical and clinical evidence in the scientific literature worldwide have supported the beneficial effects of *P. ginseng* in significant central nervous system, metabolic, infectious and neoplastic diseases. There has been growing research on *P. ginseng* polysaccharides (PGP) or *P. ginseng* proteins because of their favorable pharmacodynamics, including Qi-invigorating and anti-fatigue activity, anti-hyperglycemia and immunoregulatory activity, responsible for the efficacy of *P. ginseng* [28]. Li et al. [29] found that PGP invigorates Qi by improving energy metabolism. The current results demonstrated the hypothesis that Qi was correlated with bioenergy to a certain extent, and PGP had the pharmaceutical activities of antihypoxia, antioxidation and mitochondrial protection. *P. ginseng* has been recorded to treat “Xiaoke” (emaciation and thirst) symptoms in many ancient Chinese medical literatures [30]. “Xiaoke” generally indicates diabetes mellitus. Some researchers have extended the previous results to the chemical and pharmacological effects of another kind of active component, ginseng glycopeptide (GGP). Its hypoglycemic activity is most outstanding. The molecular weight of GGP is 6000 Da. The glycon part consists of rhamnose, arabinose, galactose, and glucose (0.46:1.61:1:2.37), and the peptide part consists of 16 amino acids (Asp, Glu, Pro, and others). There is a difference in the chemical structures of these polysaccharides, which include dextran, a type of acidic hetero-polysaccharide. The difference in the molecular weights among these polysaccharides is even greater than in the chemical structure: from Mr 1800 to Mr 1,800,000. Every ginseng polysaccharide contains a certain number of polypeptides (1.6–27%). Yet, all of these polysaccharides (100 mg kg^−1^) demonstrate anti-hyperglycemia properties [30]. *P. ginseng* polysaccharides were completely fractionated into eight fractions (two neutral fractions and six acidic fractions). Investigation of the macromolecular features revealed that the water-soluble polysaccharides contained starch-like polysaccharides, pectic arabinogalactans and RG-I-rich and HG-rich pectins. The initial bioassay indicated that ginseng polysaccharides stimulated the proliferation of both T and B lymphocytes. The neutral polysaccharides may be more potent stimulators [31]. In addition, He et al. [32] found that ginseng oligopeptides (GOP) exhibited better immunoregulatory activity compared to whey protein. The activity might be due to the increased macrophage phagocytosis capacity and NK cell activity and the enhancements in T and Th cells, as well as IL-2, IL-6 and IL-12 secretion and IgA, IgG1 and IgG2b production. *Pseudostellaria heterophylla* (*P. heterophylla*) is often used for children as a substitute for ginseng because of its mild effects [33]. *P. heterophylla* polysaccharides have been proved to benefit chronic fatigue syndrome, and the underlying mechanisms of action involve neuroendocrine and immune systems [34]. These effects may be why *P. heterophylla* is usually used as a tonic herb. A new polysaccharide named GRP (*Glehniae radix* polysaccharide) was isolated and purified from *Glehniae radix* by hot water extraction [35]. GRP is homogeneous, with a molecular weight of 1.33 × 10^4^ Da. GRP was found to be α‑D‑glucan containing (1 → 6)-linked and (1 → 3)-linked backbones with a branch of one (1 → 6)-linked and terminal glucoses at the C-4 position every fourteen residues. GRP exhibited inhibition against A549 cell proliferation and NO production in RAW264.7 cells and promoted the proliferation of mouse spleen lymphocytes and RAW264.7 cells, which suggested that GRP may have potential immunoregulatory, anti-inflammatory and anti-tumor activities. *Astragalus membranaceus* (*A. membranaceus*) is one of the most popular health-promoting herbal medicines and has been commonly used in China for more than 2000 years. *A. membranaceus* has been used historically as an immunomodulating agent for the treatment of common cold, diarrhea, fatigue and anorexia in TCM prescriptions [36]. The polysaccharides have been identified as one of the major active ingredients responsible for the above bioactivities. Niu et al. [37] extracted and purified polysaccharides (APS) from the roots of *A. membranaceus* and characterized their chemical structure and potential health properties. APS is composed of α-d-Glc residues, with the estimated equivalent dextran molecular weight of 2.07 × 10^4^ Da. APS has a repeating (1 → 4)-linked backbone with a (1 → 6)-linked branch every 10 residues. APS was able to stimulate the activity of purified mouse B cells without promoting T cell proliferation. Liu et al. [38] proved that APS was capable of improving whole-body glucose homeostasis and increasing insulin sensitivity in skeletal muscle of KKAy mice. There are three subtypes of APS:APS I, II, and III (1.47:1.21:1). APS I consists of d-glucose, d-galactose, and l-arabinose and has an average molecular weight of 36 300 kDa. Both APS II and APS III are dextrans, the linkage mode of which is mainly α-(1 → 4) linkage, and in which α-(1 → 6) linkages are exiguous. Therefore, APS has great potential for further development as products in pharmaceutical and nutraceutical areas [39]. *Rhizoma dioscoreae* (*R. dioscoreae*), also named Yam, known as an edible and medicinal tuber crop in China, has been used historically for the treatment of diabetes, diarrhea, asthma, and other ailments in TCM. Moreover, it has been consumed as a starchy food for thousands of years in China. Modern phytochemistry and pharmacological experiments have proven that non-starch polysaccharide is one of the main bioactive substances of yam [40]. YP-1 contains glucose, mannose and galactose (1:0.37:0.11). Its molecular weight was determined to be 42 kDa, and the polysaccharide has a backbone of (1 → 3)-linked α-d-glucopyranosyl residues, which occasionally branches at O-6. The branches are mainly composed of (1 → 2)-linked α-d-mannopyranosyl residues and terminate with β-d-galactopyranosyl residues. Preliminary tests in vitro revealed that YP-1 could stimulate ConA-induced T lymphocyte proliferation, and its branches are extremely important for the expression of the enhancement of the immunological activity [41]. Similarly, *R. dioscoreae* glycoproteins could promote immunity by affecting the thymus organ and phagocytic index of mice. The polysaccharide DOTP-80 was obtained by using the method of acid water extraction and ethanol precipitation. The molecular weight was calculated to be 123 kDa. The polysaccharide contains the α-configuration of sugar units and is mainly composed of mannose and glucose. A high dose of DOTP-80 (400 mg kg^−1^) had strong hypoglycemic activity [42]. Its hypoglycemic effect is the key to *R. dioscoreae* “nourishing kidney and astringent essence”. The fungal immunoregulatory protein family is effective in immunological regulation and anti-tumor activity. An immunomodulatory protein (rLz-8) was isolated from the fruiting body of *Ganoderma lucidum* (*G. lucidum*). LZ-8 is a 12-kDa polypeptide consisting of 110 amino acid residues with an acetylated amino terminus. The dose at 0.5 mg kg^−1^ of rLz-8 induced macrophage cytophagocytesis by activating the NFκB and MAPK pathways. The immune regulation of *G. lucidum* proteins is an important part of its “strengthening and strengthening the foundation”. By enhancing or regulating the immune function of the body, the damage caused by pathogenic factors on the body can be reduced, improving the resistance of the body and achieving disease prevention and treatment [43]. *G. lucidum* polysaccharide (Gl-PS) is homogeneous. The average molecular weight is 8.849 KDa, and it contains a total amount of 5.45% amino acid. The monosaccharide composition ratio of Gl-PS is glucose, galactose, arabinose, and mannose (22.4:1.9:1.0:2.1). Gl-PS could significantly decrease fasting plasma glucose, TC and TG in diabetes rats. These beneficial effects might be due to its antioxidant activities and its capacity to inhibit NO production caused by STZ [44]. Polysaccharopeptide (PSP) induced the production of serum interferon and was useful against HIV-1 infection, representing the key *Trametes versicolor* function of “clearing away heat and detoxification” [45]. Both polypeptide (PSK) and PSP could enhance immunoregulation by inducing the production of IL-6, IFNs, IgG, macrophages, and T-lymphocytes. The structure of PSK consists of a polypeptide moiety to which polysaccharide β-d-glucan chains are attached; approximately 62% of the molecule is polysaccharide, and 38% is protein. PSP is a closely related protein-bound polysaccharide, composed of 74.6% glucose, 4.8% xylose, 2.7% galactose, 2.4% fructose and 1.5% mannose. The amino acid composition is 0.58% Glu, 0.4% Asp, 0.32% Ser, 0.26% Ala, 0.26% Gly, 0.24% Leu, 0.23% Lys, 0.23% Thr, 0.22% Ile, 0.18% Arg, 0.18% Val, 0.17% Trp, 0.15% Phe, 0.15% Tyr, 0.1% Pro, 0.09% Cys, 0.07% His and 0.04% Met [46]. Licorice has the function of regulating immunity and can mediate hundreds of drugs and detoxify hundreds of poisons. At present, the material basis and mechanism of the effect of licorice on enhancing immunity are not clear. Shao et al. [47] extracted and purified licorice miRNA from licorice water extract and used the miRNA to act on peripheral blood mononuclear cells (PBMCs) and identified the utility based on cell morphological changes and cell number to reflect the effect of licorice miRNA on immune cells. Xiang et al. [48] used the miRNA extracted from licorice decoction and a synthetic licorice miRNA analog to act on PBMCs and once again confirmed that licorice miRNA had obvious immune regulation function. The results showed that licorice miRNA upregulated TLR 1 and TLR 9 expression and downregulated TLR 4 and TLR 8 expression in the TLR family. The miRNA reduced the expression of cJun and cFos, which are important components of AP-1, indicating that it may inhibit Th 2 cell differentiation by inhibiting the AP-1 pathway. Licorice miRNA also reduced the expression levels of NF-κB, p 53 and STAT 1, indicating that it may inhibit the inflammatory pathway, apoptosis and Th 1 cell differentiation. It is worth noting that the expression of the proinflammatory factor IL-6 was opposite in the miRNA group and the total extract group, indicating the complexity of the components and functions in licorice decoction. The overall effect may be caused by the interaction of licorice miRNA and secondary metabolites contained in licorice. It has been shown that compounds purified from arabinogalactan protein (containing 65% polysaccharides based on the fraction dry weight; 52% arabinose and 22% galactose are the major neutral sugars together with 6% rhamnose and 2% fucose) from *Glycyrrhiza glabra* have various biological activities, and they often act as cough suppressants. These extracts showed the capacity to reduce citric acid-induced cough in awake guinea pigs after oral administration in a dose of 50 mg kg^−1^ [49]. TCM has demonstrated that *Schisandra chinensis* (*S. chinensis*) could treat lung-Qi and kidney Yin deficiencies and relieve coughs and dementia. Polysaccharides are an important ingredient of *S. chinensis* (SCP) and often appear in ancient prescriptions for forgetfulness or dementia [50]. SCP could improve the cognition of mice, and it may play an anti-AD role by activating the NF-κB/MAPK pathway to alleviate neuroinflammation [50]. Chi et al. [51] found that SCP is a protein-bound polysaccharide consisting of 12 amino acids. SCP has a therapeutic effect on chronic fatigue syndrome that is partially related to TCA cycle metabolic pathways and alanine, aspartate and glutamate metabolism. Zhong et al. [52] found a new polysaccharide (SCFP-1). The molecular weight is 3.18 × 10^4^ Da, and it is mainly composed of glucose and arabinose (66.5% and 29.4%, respectively). Peroral administration of SCFP-1 at 250, 500, and 1000 mg kg^−1^, respectively, showed remarkable suppressive effects on both chronic and acute cough.

*Angelica sinensis* (*A. sinensis*) polysaccharide (ASP) is an important bioactive component for the hematopoietic effect of *A. sinensis* that has been used in TCM for treating anemia and gynecological disorders. ASP also inhibited the expression of signal transducer and activator of transcription 3/5 and mothers against decapentaplegic proteins 4 in liver and stimulated the secretion of erythropoietin; it could also be applied in the treatment of anemia. ASPS contains two subtypes, ASP I and II (7.41:1), both of which consist of arabinose, galactose, and glucose. ASPS suppressed hepcidin expression in vivo by stimulating erythropoietin secretion and interrupting the other 2 main pathways of hepcidin regulation. This finding backed the speculation that ASP could participate in the regulation of iron homeostasis [53]. Pan et al. [54] found that *A. sinensis* protein could significantly promote normal human hepatocyte proliferation, making its cell viability as high as 655%, while it has a significant inhibitory effect on leukemia cells, reducing cell viability to approximately 80%, which is similar to the “righting and dispelling evil” argument in Chinese medicine. *Radix Rehmanniae Preparata* (*R. Rehmanniae Preparata*) is the prepared root of *Rehmannia glutinosa* (*R. glutinosa*). *R. Rehmanniae Preparata* is used for nourishing Yin and tonifying the kidney and has the functions of storing essence; dominating growth, development and reproduction; and regulating water metabolism in the body according to TCM theory. Modern research has also indicated that polysaccharides are the main chemical components related to bioactivities and pharmacological properties. The contents of *R. Rehmanniae Preparata* polysaccharides (RRPP) in different habitats ranged between 0.98% and 5.09%. Research has shown that there are two acidic polysaccharides, called rehmannan SA and rehmannan SB, in RRPP. They are commonly composed of l-arabinose, d-galactose, l-rhamnose, d-galacturonic acid in the molar ratios of 10:10:1:1 (rehmannan SA) and 14:7:3:8 (rehmannan SB). RRPP may be responsible for the pharmacological effect of anti-fatigue. The mechanism is related to the increase of the storage of hepatic glycogen and the decrease of the accumulation of SUN and BLA [55]. According to reports, RRPP can also stimulate hemopoiesis, immune enhancement, and anti-diabetes effects [55].

Maidong is used to nourish Yin, moisten the lungs, clean the heart-fire, relieve the drought of mouth and tongue (Xiaoke syndrome), and treat vexation, insomnia and cough [56]. Polysaccharides, the main composition of Maidong, with an extraction rate up to 35% [57], have attracted great attention in the carbohydrate polymers field. *Liriope spicata* polysaccharide (LSP), *Ophiopogon japonicus* polysaccharide (OJP) and *Liriope muscari* polysaccharide (LMP) are composed of β-fructose and α-glucose. The average molecular weights of LSP, OJP and LMP are 4742, 4925 and 4138 Da, with polydispersity indexes of 1.1, 1.2 and 1.1, respectively. The backbones of the polysaccharides are formed by Fruf-(2 → , 2 → 2)-Fruf-(6 → , → 6)-Glcp-(1 →  and  → 1,2)-Fruf-(6 → , with molar ratios of 5.0:18.2:1.0:5.3 (LSP), 6.8:15.8:1.0:5.8 (OJP), and 8.3:12.3:1.0:3.9 (LMP), respectively. LSP, LMP and OJP increased the expression of PI3K, AKT, InsR, and PPARγ and decreased the expression of PTP1B at both the mRNA and protein levels in IR HepG2 cells. Furthermore, glucose consumption was increased after treatment with polysaccharides. These results revealed that LSP, OJP and LMP had potential anti-diabetic effects [58]. The fruit of *Lycium barbarum* (*L. barbarum*), also called Goji berry or wolfberry, is a well-known TCM and a valuable nourishing tonic. Recently, it has also been widely marketed as a health food and anti-aging remedy in Western countries. As an immunoregulatory, anti-diabetic and anti-senile agent, wolfberry plays an important role in preventing and treating various chronic diseases, such as diabetes, hyperlipidemia, immunodeficiency, and male infertility [59]. Luo et al. [59] found that crude LBP and the purified polysaccharide fraction (LBP-X) could reduce the blood glucose levels and serum TC and TG concentrations while increasing HDL cholesterol levels. LBP-X contains six monosaccharides, including rhamnose, galactose, glucose, arabinose, mannose, and xylose, in a molar ratio of 4.22:2.43:1.38:1.00:0.95:0.38. Amino acid analysis has revealed that LBP-X contains 17 amino acids. The total content of amino acids was 8.46%. Peng et al. [60] and Peng and Tian [61] also isolated five glycoconjugates and elucidated their structures (LbGp1-LbGp5); they are mainly composed of two to six monosaccharides and 17 amino acids. The molecular weights of these isolated glycoconjugates range from 23.7 to 214.8 kDa. Both of them possess immunomodulatory activities with different mechanisms. Chen et al. [62] reported that a polysaccharide-protein complex isolated from *L. barbarum* can activate macrophages. LBP was isolated and separated into five homogenous fractions (LBPF1-5). It was found that LBP enhanced innate immunity by activating macrophages by the activation of the transcription factors NF-κB and AP-1 to induce TNF-α production and upregulation of MHC class II costimulatory molecules. Additionally, LBP attenuates diabetic testicular dysfunction via inhibition of the PI3K/Akt pathway-mediated abnormal autophagy in male mice [63]. This result is related to the tonifying kidney and essence effect of *L. barbarum*. *Alpiniae oxyphyllae* fructus (*A. oxyphyllae*) can warm the kidney, reduce the urine and store essential substances according to TCM theory. *A. oxyphyllae* has been widely used to treat urinary incontinence (UI) in Chinese medicine [64]. *A. oxyphyllae* polysaccharides (PAOF), with a molecular weight of 278 kDa, have many physiological activities closely related to the treatment of UI, including immunoregulation, anti-inflammation, and antioxidation. PAOF can significantly reduce the urination volume, Na^+^/Cl^−^ emission and increase K^+^ excretion in hydruric model rats (OHMR). In addition, PAOF can increase aldosterone and antidiuretic hormone contents in the blood of OHMR. The coefficients of spleen, thymus and adrenal of OHMR were improved by PAOF. Furthermore, PAOF can not only significantly increase the expression of β3-adrenoceptor mRNA in bladder detrusor of OHMR but also increase the contents of adenylate cyclase (AC), cyclic adenosine monophosphate (cAMP) and protein kinase A (PKA) in bladder detrusor of OHMR. Meanwhile, PAOF can significantly elevate the expression of PKA protein in bladder detrusor of rats with polyuria. The data implied that PAOF may offer therapeutic potential against UI [64].

*Cordyceps sinensis* (*C. sinensis*) is a precious and highly regarded medicinal fungus in TCM with a broad range of health benefits, such as improving liver, kidney, lung, and immune functions [65]. Polysaccharides are well documented to be major active ingredients of *C. sinensis*, ranging from 3 to 8% of the total weight and usually coming from the fruiting bodies, the mycelium of solid substrate fermentation, and the liquid broth [66]. More recent studies suggest that polysaccharides from cultured *C. sinensis* possess great potential biological properties, such as immunomodulatory [67], hypoglycemic [68], and anti-fibrosis functions [69]. Recently, researchers have taken more interest in studying the chemical structures and biological activities of polysaccharides from cultured *C. sinensis* [70]. The chemical structure, chain conformation, and immunomodulatory activity of polysaccharide from mycelium *C. sinensis* fungus UM01 were investigated. The molecular weight and the intrinsic viscosity of purified polysaccharide (UM01-S4) were determined to be 22,559 Da and 5.09 mL g^−1^, respectively. The chemical structure of UM01-S4 contains an α-(1 → 2)-Manp core. The side chains are composed of β-(1 → 2)-Galf, β-(1 → 4)-Glcp, α-GalpA, and α-Manp units, which are attached to the mannan core at the O-6 position. The immunomodulatory assays showed that UM01-S4 stimulated macrophage proliferation, phagocytosis, and release of NO and cytokines. The mechanism underlying the macrophage regulation of UM01-S4 might be related to the activation of the MAPK and NF-κB signaling pathways [65]. A water-soluble polysaccharide (CPS-2), isolated from cultured *C. sinensis*, was obtained by hot-water extraction. CPS-2 was found to be mostly α-(1 → 4)-d-glucose and α-(1 → 3)-d-mannose, branched with α-(1 → 4,6)-d-glucose every twelve residues on average. CPS-2 has a molecular weight of 4.39 × 10^4^ Da. The protective effect of CPS-2 on the model of chronic renal failure was established by fulgerizing kidney. The changes in blood urea nitrogen and serum creatinine revealed that CPS-2 could significantly relieve renal failure caused by fulgerizing kidney [71]. *Pilose antler* (*P. antler*) is a “warm kidney and aphrodisiac” medicinal material commonly used in TCM, and it has significant improvement and promotion effects on gonadal function. He et al. [72] found that the *P. antler* polypeptide (consisted of 34 amino acids) may be one of the effective ingredients that affect sexual function. The *P. antler* polypeptide may directly act on pituitary cells to promote the release of LH and T and inhibit the release of PRL. An immunomodulatory protein (PCP) (35.6 kDa) is a disulfide-linked heterodimeric glycoprotein consisting of 14.3 and 21.3 kDa subunits with *N*- and *O*-glycosylations [73]. PCP stimulated mouse peritoneal macrophages (RAW264.7) by interacting with toll-like receptor 4 and subsequently activating the NFκB signaling pathway. Oral administration of PCP reduced the production of serum total IgG1 and OVA-specific IgG1 and upregulated the serum OVA-specific IgG2a and splenic Th1-related cytokine and downregulated IL-4 and IgE levels in atopic dermatitis mice [74]. *P. antler* tray proteins (PSAB) is a mixture of 5 proteins with a molecular mass of 10 to 70 kDa and includes 17 amino acids. PSAB enhanced the anti-fatigue effect and adrenal function of the body, protected paint-stressed mice, and increased the number of red blood cells and the hemoglobin content [75]. *Semen cuscutae* (*S. cuscutae*) is a well-known Chinese medicine that has been used to nourish the kidney. A study demonstrated that the polysaccharides from *S. cuscutae* showed significant activity of nourishing kidney Yang by increasing the levels of testosterone and estradiol, decreasing the level of blood urea nitrogen, and improving immune function; in addition, it possessed an antioxidant effect [76]. Three homogeneous polysaccharides were obtained and named C-7WR1, C-7WR2 and C-7WR3, with average molecular weights of 7.59 × 10^4^, 3.23 × 10^4^ and 2.25 × 10^4^ Da, respectively. C-7WR1 is composed of fructose and mannose (0.02:1). C-7WR2 is composed of fructose, mannose, xylose, and arabinose (0.01:1:0.14:0.33). C-7WR3 is composed of fructose, mannose, xylose, and arabinose (0.01:1:0.10:0.47). They mainly contain mannose and have no nucleic acid or protein. Moreover, their Fourier transform infrared features were similar [76].

Antipyretics generally have anti-inflammatory and antiviral effects, along with related effects. The functions of “Clearing heat and detoxifying” in *Prunellae Spica* (*P. Spica*), *Lonicera japonica* (*L. japonica*) and *Trichosanthis radix* (*T. radix*) were demonstrated by *P. spica* polysaccharide (PSP-2B), *L. japonica* miRNA (miR2911) and *T. radix* proteins (TCS) with antiviral activity. *L. japonica*, a well-known Chinese herb, has been used to effectively treat influenza infection for thousands of years. Several reports have shown that its decoction can suppress the replication of influenza virus [77]. The present study of Zhou et al. [78] provided the first evidence that the highly stable plant miR2911 can directly target multiple viral genes of various IAVs and thus suppress viral infections. With its broad-spectrum anti-viral activity against IAVs, miR2911 and miR2911 contained in *L. japonica* decoction may represent an effective new therapeutic strategy that can be used to subdue deadly IAV infections. *T. radix* proteins (TCS) preferentially inhibited the replication of human immunodeficiency virus type 1 (HIV-1) in both acutely infected T-lymphoblastoid cells and chronically infected macrophages in vitro [79]. TCS was found to decrease the serum HIV-1 p24 antigen level and to increase the percentage of CD4C cells in patients with acquired immunodeficiency syndrome (AIDS) and AIDS-related complex [80]. Initially, it was believed that the anti-HIV activity is related to its ribosome-inactivating activity. However, the same group later found that two TCS variants with a 19-amino-acid extension and a KDEL signal sequence added to the C-terminal sequence, respectively, lost most of their anti-HIV activity without losing ribosome-inactivating activity [81]. As a protein, TCS elicits antibody responses such as IgE and IgG production, which limit its multiple administration. Anaphylactic responses appeared after the injection of TCS in HIV-infected patients. The anaphylactic reactions may be due to the activation of complement by TCS via the alternative pathway. TCS also displays selective immunosuppressive actions. Non-cytotoxic concentrations of TCS suppress the activation, multiplication and differentiation of T and B cells but do not suppress the activation of natural killer cells [82]. Hence, TCS has a complicated interaction with the immune system. Further work on this aspect is needed to elucidate the mechanism and to minimize the side effects. A novel polysaccharide, PSP-2B, was isolated from aqueous extracts of *Prunellae Spica*. PSP-2B is a partially sulphated polysaccharide with a molecular weight of approximately 32 kDa. Its sulfate content was determined to be 10.59% by elemental analysis. The major sugars comprising PSP-2B were arabinose, galactose and mannose, in addition to small amounts of glucose and uronic acids. PSP-2B also contains 2.98% protein. PSP-2B exhibited activity against herpes simplex virus (HSV), with half maximal inhibitory concentrations (IC50s) of approximately 69 g mL^−1^ for HSV-1 and 49 mg mL^−1^ for HSV-2. However, PSP-2B demonstrated no cytotoxicity, even when its concentration was increased to 1600 mg mL^−1^, suggesting that it has potential as an anti-HSV drug candidate [83]. *Anemarrhena asphodeloides* (*A. asphodeloides*) possessed the effects of nourishing Yin, moistening dryness, clearing lungs and relieving fire. Simultaneously, it has been used to treat constipation for more than one thousand years in China. Recent findings suggested that *A. asphodeloides* polysaccharides (AABP), consisting of d-mannose, l-rhamnose, d-galacturonic acid, d-glucose, d-galactose and l-arabinose (1:0.04:0.53:0.11:0.33:0.25), with an average molecular weight of 1.11 × 10^3^ kDa, have an active laxative function, which could treat constipation by regulating the gastrointestinal hormones and neurotransmitters to improve the intestinal motility and water metabolism [84]. Moreover, polysaccharides are supplementary therapeutic agents for constipation and have been indexed by the FDA [85]. *Rehmannia glutinosa* (*R. glutinosa*) has been widely used as Chinese medicine for the treatment of diabetes and its complications. The *R. glutinosa* polysaccharide fraction (RGP) has been proposed to possess a hypoglycemic effect by intraperitoneal administration. RGP is composed of rhamnose, arabinose, mannose, glucose and galactose in the molar ratio of 1.00:1.26:0.73:16.45:30.40, with an average molecular weight of 63.5 kDa. RGP administration significantly decreased the blood levels of glucose, total cholesterol, triglycerides, and low-density lipoprotein cholesterol and increased the blood levels of high-density lipoprotein cholesterol and insulin in diabetic mice, concurrent with increases in body weight and pancreatic insulin content. Moreover, RGP reversed the increased mRNA expression of PEPCK and reduced glycogen contents in the livers of diabetic mice [86]. Huang et al. [87] found that RGP significantly stimulated lymphocyte proliferation, and the T cell growth rate was even more significant. The IL-2 and IFN production levels of T lymphocyte were significantly upregulated after being stimulated with RGP. A cholesterol-lowering protein from *Senna obtusifolia* seeds was used in TCM to treat hyperlipidemia and hypertension and to remove liver heat (gan re). This cholesterol-lowering protein is a single protein with a molecular weight of 19.7 kDa and a pI of 4.8 [88]. The N-terminal amino acid sequence of this peptide has no homology with any other protein sequences in GenBank. Its secondary structure has 12.5% α-helix, 55.6% β-sheet, and 31.9% random coil properties. This cholesterol-lowering protein inhibited cholesterol biosynthesis in Chinese hamster oocytes [88]. Earthworm is used to treat cerebrovascular diseases in TCM. Nakajima et al. [89] reported that extracts of an earthworm, *Lumbricus rubellus*, contained six different fibrinolytic isoenzymes (EFEs). EFE-III-1 and III-2, two of the isomers, had strong fibrinolytic activities, broad pH optima (pH 9–11) and resistance to thermal and guanidine-HCl denaturation [90]. These features were effective and useful for EFEs to treat some clotting diseases. Mihara et al. [91] found that the enzymes activated the endogenous fibrinolysis system by oral administration, with a significant fibrinolytic effect on clots in blood vessels [92].

Blood is one of the important substances of the human body, but it must be free and smooth to nourish the whole body. If it is blocked, pain, mass and other diseases often occur. The functions of blood-activating and stasis-removing drugs are to disperse blood stasis and to relieve all kinds of diseases caused by blood stasis block. Thus, the clinical application is very important. *Salvia miltiorrhiza* (*S. miltiorrhiza*) is a TCM in the treatment of many diseases, especially ischemic cardiovascular diseases. According to Chinese medicine theory, *S. miltiorrhiza* promotes blood flow and resolves blood stasis. Song et al. [93] purified and partially characterized a homogenous polysaccharide SMP1 fraction from the roots of *S. miltiorrhiza*. The average molecular weight of SMP1 is 5.5 × 10^5^ Da. The monosaccharide composition is a heteropolysaccharide consisting of galactose, glucose, fucose, rhamnose, arabinose and mannose in a relative molar ratio of 1.0:1.2:0.3:1.5:1.3:1.9. SMP1 contains 91.3% total carbohydrate, 2.81% uronic acid and 4.34% protein. The protective effect of SMP1 on myocardial ischemia–reperfusion (I/R) injury has been studied. Pretreatment with SMP1 (400 and 800 mg kg^−1^) one week before the ligation of the LAD coronary artery caused a significant reduction in infarct size in I/R rats. Moreover, the increases in the levels of serum LDH, serum CK and myocardial MDA and the decreases in the myocardial SOD, Na^+^-K^+^-ATPase and Ca^2+^-Mg^2+^-ATPase activities in I/R rats were reversed by oral administration of SMP1 at doses of 400 and 800 mg kg^−1^. Moreover, a TUNEL assay indicated that SMP1 could suppress cardiocyte apoptosis [94]. *Achyranthes bidentata* (*A. bidentate*) has been traditionally used in China as a natural remedy for osteoporosis. The crude *A. bidentate* polysaccharide ABW70-1 is composed of (2 → 1)-linked-β-d-fructofuranosyl (Fruf), (2 → 6)-linked-β-d-Fruf and (2 → 1,6)-linked-β-d-Fruf residues, terminating with fructose and glucose residues. ABW70-1 stimulated the osteogenic differentiation of MC3T3-E1 cells by promoting cell proliferation, ALP activity, mineral nodule formation and the gene expression of OSX, OCN and BSP [95]. These results suggested that *A. bidentate* polysaccharides have great potential in the prevention and treatment of osteoporosis. A novel polysaccharide (ABPB-3) was purified from *A. bidentata* polysaccharide (ABPB), and its structure was characterized as a repeating unit consisting of  → 4)-α-d-GalpA-(1 → , → 2,4)-α-l-Rhap-(1 → , → 5) -α-l-Araf-(1 → , → 2,3,5)-α-l-Araf-(1 → , → 3)-β-d-Galp-(1 → , → 3,4,6)-β-d-Galp-(1 → , terminated with α-l-Araf, α-l-Rhap and β-d-Galp. In the zebrafish model of glucocorticoid induced osteoporosis, ABPB-3 significantly increased the relative fluorescence intensity of the skull bone mass in a concentration-dependent manner, indicating that it stimulated bone formation activity [96]. Thus, ABPB and ABPB-3 have the potential to be used for anti-osteoporosis medicine. Zhang and Lin [20]. demonstrated that ABP had clear anti-physical fatigue effects that could extend the exhaustive swimming time of the mice, increase the liver glycogen and muscle glycogen contents and decrease the blood lactic acid and blood urea nitrogen contents. These observations fit the effects of *A. bidentate* on tonifying the liver and kidney, strengthening the muscles and bones.

*Gastrodia elata* (G. *elata*) is mainly used for the treatment of infantile convulsions, nervous headaches, epilepsy, hypertension and other diseases. *G. elata* polysaccharide (PGE) has a molecular weight of 1.54 × 10^3^ kDa. The total polysaccharide content of PGE is 94.27%. The optical rotation of PGE is + 155°. The monosaccharide is mainly composed of glucose. Furthermore, the backbone of PGE is composed of (1 → 4)-linked-d-Glcp and the branches of (1 → 3)-linked-d-Glcp, (1 → 4,6)-linked-d-Glcp and (1 →)-linked-glucose termini. PGE is a high-molecular-weight polysaccharide that exhibits angiotensin-I converting enzyme (ACE) inhibitory activity. Its inhibition rate on ACE was calculated as 74.40%, and the IC50 value was 0.66 mg/mL [97]. The purified *G. elata* polysaccharide (PGEB-3H), was found to be a glucan with a molecular weight of 28.8 kDa and specific rotation of + 206.3°. PGEB-3H is mainly composed of glucose and has a (1 → 4)-α-d-glucan main chain occasionally branched with α-1,6-glycosidic linkages. PGEB-3H exhibited potential lipid-lowering effects in hyperlipidemia rats [98].

Stated thus, polysaccharides, proteins, and miRNAs of TCM play an indispensable role in the development of their pharmacodynamics-related biological activities. Such studies are mainly focused on tonic, antipyretic and blood-activating drugs. However, herbs are generally boiled for several hours to prepare the decoction. It is commonly believed that proteins and RNA will be destroyed during this process. Most current studies of proteins or peptides have overlooked that fact. Indeed, some data showed that certain miRNAs, such as miR2911, was found to be largely intact in the final decoction. However, the mechanism underlying the high stability of miR2911 during the boiling process remains unknown. Zhou et al. [78] indicated that its unique sequence and high GC content may contribute to its stability. The resistance of miR2911 to boiling processes or even RNAse treatment was abolished after the sequence was changed and the GC content was decreased. The results reveal that miRNA may be an important, potentially effective but previously unrecognized component in Chinese herbs. Therefore, how polysaccharides, proteins or miRNAs in TCM therapeutically contribute after traditional oral administration warrants further investigation.

### Action mechanisms of biomacromolecules in Chinese medicine

#### Mechanisms of action of Chinese medicine polysaccharides

Most polysaccharides are directly discharged from urine in the body, and the components can be absorbed and are degraded into monosaccharides or oligosaccharides. The oligosaccharides produced in the process of polysaccharide hydrolysis will interact with the body’s immune system. Activities such as binding with the cellular receptor on the mucous membrane to activate signaling pathways and cause an immune response, ultimately playing a therapeutic role. There has also been some research on the relationship between polysaccharides and their receptors. The difference in immune activity of polysaccharides may be related to the different receptors on the immune cells’ surfaces or a combination of different factors [99]. Some scholars also studied the polysaccharide active fragments (active determinants) from *Bupleurum*, *Angelica* polysaccharides and lentinan. They believe that polysaccharides, just like proteins and enzymes, may have one or several “active sites” of oligosaccharide fragments that can combine with the receptors of immune cells to activate the immune system [100]. Modern network immunity doctrine says that after receiving stimulation from antigen, immunological regulation generates the immune response, including mutual promotion and interaction among a variety of immune cells and immune molecules [101]. During the process, the participation of genetic regulation and the nervous system is needed. As a type of biological response modifier (BRM), polysaccharides act mainly by activating the host immune system, including innate and adaptive immunity, with anti-tumor, anti-virus and anti-aging functions. Polysaccharides can activate macrophages, T lymphocytes, B lymphocytes, NK cells, cytotoxic T cells (CTL), and lymphokine-activated killer cells (LAK) and promote the production of cytokines and antibodies and activate the complement system [102]. Through these pathways, polysaccharides can achieve multilevel, multichannel, multitarget regulation of the immune system. Overall, they are related to the regulation of the neuroendocrine-immune (NEI) network. At the molecular level, polysaccharides can combine with receptors on the surfaces of immune cells such as macrophages to initiate the immunoregulatory effect, change the levels of second messengers (i.e., the concentration of NO, Ca^2+^, cAMP and cGMP), and then induce the production of cytokines to carry out cell signaling, thus playing a series roles in immune regulation [101].

As mentioned in the first section, the biological activities of polysaccharides are known to be closely correlated with their chemicophysical properties, such as monosaccharide composition, features of glycosidic linkages, molecular weights, and chain conformations. The solubility of polysaccharides directly affects their hydrolysis and absorption and then influences their biological effects. Most of the polysaccharides are water-soluble heteropolysaccharides, some of which contain acidic groups (galacturonic acid) or proteins, which affect their solubility and activity. Polysaccharides with too-large relative molecular weights are disadvantageous to cross the cell membrane into the human body to exert biological effects, and polysaccharides with too-small relative molecular weights have no activity [103]. As seen in Table 1, the molecular weights of polysaccharides vary from a few to tens of thousands of kDa. For instance, the differences in the molecular weight among ginseng polysaccharides are even greater than the differences in chemical structure. Yet, all of these polysaccharides are effective in anti-hyperglycemia. The sugar unit composition and glycosidic bond type of the main chain of Chinese medicine polysaccharides directly determine the activity, branch chain type and polymerization degree of polysaccharides. The distribution and the degree of substitution on the polysaccharide chain determine the activity of Chinese medicine polysaccharides, and the higher structure of Chinese medicine polysaccharides has more influence on its activity [104]. *Radix Rehmanniae* (*R. Rehmanniae*) polysaccharides (RGP) consist of rhamnose, arabinose, mannose, glucose and galactose and exhibit anti-diabetes activity, while *R. Rehmanniae* Preparata polysaccharides (RRPP) are commonly composed of l-arabinose, d-galactose, l-rhamnose, d-galacturonic acid and exhibit anti-fatigue activity. Fungal polysaccharides have a high anti-tumor function; higher plant polysaccharides can enhance immune regulation best; and algal polysaccharides with sulfuric acid have better anti-clotting and anti-viral effects [105]. For example, the main medically important polysaccharides that have undergone extensive anticancer clinical trials include lentinan (from *Lentinula edodes*), schizophyllan (from *Schizophyllum commune*), Grifron-D (from *Grifola frondosa*), PSK (polysaccharides-K, commercially sold as Krestin) and PSP (polysaccharopeptide, from *Trametes versicolor*). The first three are β-(1,3)-d-glucan compounds. However, α-glucans (e.g., starch, cellulose and chitin) have no biological activity. The macromolecular structure of β-glucans depends on both the source and method of isolation, varying mainly in the distribution and length of side chains, which provide for complex tertiary structures stabilized by inter-chain hydrogen bonds. Some polysaccharides of Chinese medicine can enter intestinal cells with the help of catarrh proteins, induce favorable changes in the intestinal microbiota and play a variety of biological activities [3]. Multiple indigestible dietary carbohydrates, such as oligofructose, galactooligosaccharides, lactulose and inulin (long-chain β-fructan), are proven to be prebiotics that selectively stimulate the growth of a subset of beneficial gut bacteria, and consequently to maintain the homeostasis of gut microbial community as well as the host health [106]. It has also been fully evidenced that various diseases, such as obesity, diabetes and cancer, may change the compositions of gut microbiota [107] and that both the pathological symptoms and the gut microbiota dysbiosis can be alleviated by TCM, including TCM polysaccharides, although such functional connections are still less well understood [108]. These facts described above indicated that polysaccharides in TCM decoction, although indigestible when orally administered, potentially work directly (as prebiotics) and/or indirectly (under certain pathological conditions) to induce favorable changes in the intestinal microbiota. The improved gut microbiota then further enhances intestinal metabolism and absorption of the bioactive small molecular chemicals co-administered in the TCM decoction. Zhou et al. [3] provided a novel gut microbiota-involved mechanism by which polysaccharides synergistically work with small molecular chemicals co-existing in TCM decoction on the pathological model. The facts suggest that TCM polysaccharides, even indigestible by the host, could still indirectly contribute to the therapeutic effects of TCM decoction.

#### Mechanisms of action of Chinese medicine proteins

**Fig. 1:**
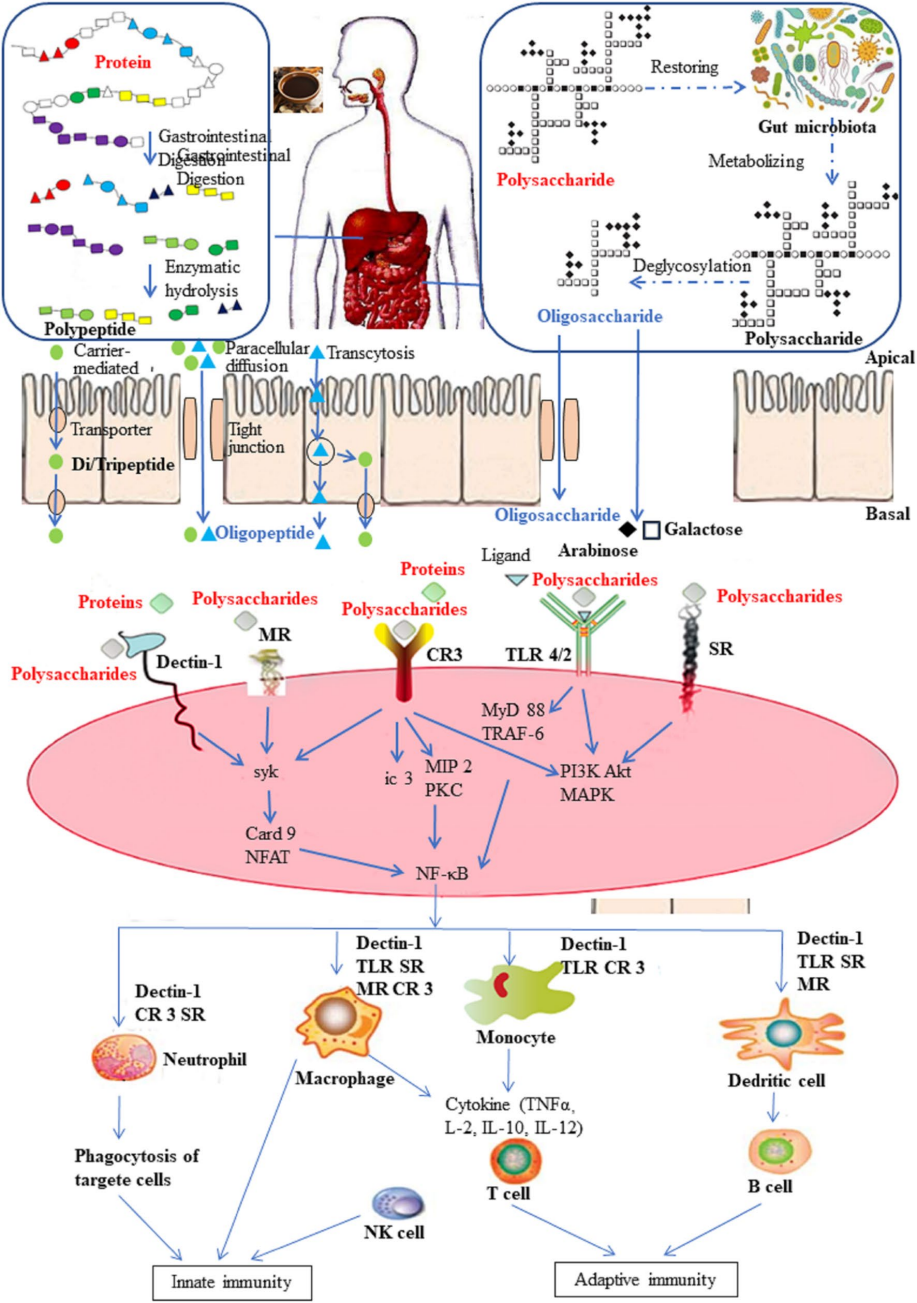
Absorption, metabolism and immunoregulatory effects of polysaccharides and proteins. *Card 9* caspase recruitment domain family, member 9; *CR3* complement receptor 3; *iC3* immune component 3; *MIP2* macrophage inflammatory proteins 2; *MR* mannose receptor; *MyD 88* myeloid differentiation primary response gene (88) ; *NFAT* nuclear factor and activator of transcription; *SR* scavenger receptor; *syk* spleen tyrosine kinase; *TLR* toll-like receptor; *TRAF-6* TNF-receptor-associated factor 6

As TCM and modern western medicine share a common aspect at the molecular level that the compounds perturb the human’s dysfunction network and restore human normal physiological condition. The relationship between compounds (in herbs, refer to ingredients) and their targets (proteins) should be the key factor to connect TCM and modern medicine. Therefore, proteins have long been studied as targets. Recently, increasing numbers of bioactive proteins with various pharmacological properties have been successfully isolated from animals such as *Hirudo medicinalis*, *Eisenia fetida*, and *Mesobuthus martensii*, and from herbal medicines derived from species such as *Cordyceps militaris*, *Ganoderma*, *Poria cocos*, *Senna obtusifolia*, *Dioscorea batatas*, and *Trichosanthes kirilowii*. Moreover, some protein pharmaceuticals from Chinese medicine have been developed to treat cardiovascular diseases, genetic diseases, and cancer. However, protein drugs can be rapidly eliminated in vivo, which is due to a large amount of protease in tissues, which can hydrolyze protein drugs and make them lose their activity. At the same time, due to the filtration effect of glomeruli, molecules with molecular weights of less than 69 kDa are easily removed in the process of metabolism. Therefore, there has been some doubt whether the protein will be digested by the stomach and intestines like meat when it enters the human body. He et al. [92] revealed the process of absorption of Lumbrokinase into the blood in the whole digestive tract. They found that the spatial structure of Lumbrokinase is particularly easy to stretch; thus, it has a strong specificity. After oral administration, the whole protein molecule is absorbed, and some enzymes are released into the blood through intestinal epithelial cells, which degrade coagulation factor 1 to achieve the purpose of treatment and the prevention of cerebral thrombosis. They also determined that Lumbrokinase, when absorbed into the bloodstream, can be bound to and inhibited by α2M macroglobulin. The chemical structure of a protein determines its activity. The main structural factors affecting the activity are the amino acids and their sequence, terminal groups, peptide chains and disulfide bond positions. The two- and three-dimensional structures of drugs also affect the biological activity. Moreover, the molecular weights of polypeptides and proteins are usually in the thousands to hundreds of thousands, and the particle sizes range between 1–100 nm; thus, they cannot penetrate the semipermeable membrane. LZ-8 is a 12-kDa polypeptide consisting of 110 amino acid residues with an acetylated amino terminus. The crystal structure of LZ-8 supplies a basis to study its bioactive function. The C-terminal FNIII domain possesses the immunoglobulin-like β-sandwich fold to recognize its targets, including cytohormones, cell adhesion molecules, cytokine receptors, molecular chaperones and carbohydrate-binding domains [109]. The molecular mass of GCL (18 kDa) is near the lower end of the range (15 to > 100 kDa) reported for mushroom lectins. The specificity of lectin toward galactose/galactosamine is similar to those of lectins from *Agrocybe aegerita*, *Fomes fomentarius*, *Psilocybe barrarae*, and its N-terminal sequence displays slight similarity to a lectin from *Ganoderma lucidum* and fungal immunomodulatory proteins from *Flammulina velutipes* and *Volvariella volvacea* [110]. The results have shown that the main forms of Chinese medicine proteins function in determining their efficacy, including: (1) Taking extracted and purified proteins as the medicinal portion, such as TCS, with physiological functions of immune regulation, inhibited the replication and reproduction of human HIV [111]. (2) Taking glycoproteins as the active component, it has been shown that compounds purified from arabinogalactan protein from *Glycyrrhiza glabra* have various biological activities, such as antioxidant, anti-inflammatory, immunomodulating, antispasmodic action, or antiallergic properties, and they often act as cough suppressants. (3) Polysaccharide peptides are the active components obtained from protease hydrolysis. A considerable portion of the polysaccharide exists in the form of glycoprotein, most of which have widely ranging bioactivity. For example, ginseng glycopeptide has hypoglycemic activity and immunoregulatory activity; *Rhizoma dioscoreae* glycoproteins have immunoregulatory activity; and *Schisandra Chinensis* glycopeptides have anti-fatigue activity. (4) As the active ingredient in the form of peptides, including the peptide compounds extracted and purified. For instance, hirudin, the most powerful thrombin natural specific inhibitor discovered thus far, is from leech, a TCM, and is an effective anticoagulant and antithrombotic [112]. Another kind of peptide from Chinese medicine has no activity in the original protein sequence, but it can show pharmacological activity after being hydrolyzed by protease, with the hydrolyzed peptide functioning as the active site. Furthermore, compared with native proteins, small peptides show higher antioxidant capacity and can be absorbed in the gastrointestinal tract without further digestion. In addition, stronger activities of proteins have been found in components with molecular weights of less than 1 kDa (2–10 amino acid residues, oligopeptides). *Panax ginseng* oligopeptides could enhance innate and adaptive immune responses in mice. Modern research also demonstrated that proteins are mostly in the form of oligopeptides when they are absorbed by the body, and these oligopeptides may be the effective components of Chinese medicine proteins [113]. Although it is known that the protein components of TCM and the hydrolyzed oligopeptide components can play a pharmacological role, the material basis of the efficacy of Chinese medicine proteins, the characteristics of the structure of hydrolyzed oligopeptide components, and the mechanisms of their efficacy are still unclear. This review summarizes the mechanisms of polysaccharides, protein absorption, metabolism and immune regulation, as shown in Fig. 1.

#### Mechanisms of action of Chinese medicine miRNAs

In vivo and in vitro analyses and numerous scientific evidence have shown that the nucleic acids of TCM can transfer into the cells of the body through the gastrointestinal tract and translate or express products with specific biological functions [114]. In addition to the confirmation that foreign miRNA can reach the blood and digestive tract, trace amounts of foreign miRNA have been found in the heart, liver, kidney and breast. Additionally, some miRNAs in *Lonicera japonica, Glycyrrhiza uralensis* and other TCM can enter the human body through oral water decoction and show significant biological activity and tissue targeting. However, the specific mechanism of miRNA is not clear, mainly due to the formation of a complex interaction network between an miRNA and its target mRNA. For example, an miRNA can bind and regulate a variety of mRNAs, and at the same time, an mRNA can bind to multiple miRNAs; thus, the mechanism of action is complex and diverse. The specific mechanism employed by miRNA is still a scientific problem to be solved, and the research on miRNA regulatory networks is still in its infancy.

### Biological function of biomacromolecules in medicinal plants

#### The role of biomacromolecules in the growth and development of medicinal plants

As the first defense layer of plants, the cell wall, approximately 90% of which is composed of polysaccharides (the monosaccharide units that make up these polysaccharides are mainly β-d-glucose, galacturonic acid, mannose and arabinose), plays an important role in responses to biotic and abiotic stresses, and sugar is the main factor that achieves this function [115]. More than 1/3 of the proteins in organisms are glycoproteins that play important roles in seed germination, seedling growth, reproduction and various stresses. Examples include hydroxyproline-rich glycoproteins (related to plant-induced resistance) and arabinogalactan proteins (which play a role in the fertilization process of angiosperms). In particular, cell surface glycoproteins are often the first receptors to receive external signals and are responsible for the information exchange between cells and the outside world; thus, scientists are paying increasing attention to the functions of cell surface glycoproteins [22]. Furthermore, it has been found that oligosaccharides have good control effects on more than 100 diseases in ten crops. Some research has shown that resistance could be achieved mainly by activating plant autoimmunity, which is the basis of the proposed concept of oligosaccharide plant vaccines [116]. The action mechanisms of oligosaccharide plant vaccines in plants may be as follows: sugar signal is transferred into the cell through the recognition receptor on the membrane and a series of signal transduction events. Then, the expression of related defense genes are regulated, promoting the accumulation of secondary metabolites of resistance, inducing the generation of the resistance response, and ultimately effectively resisting pathogen infection [117]. On this basis, scientists also found that many sugars can promote plant growth, improve product quality and resist stresses.

The biological functions of proteins in medicinal plants can be summarized as follows: (1) the catalytic functions of enzymes. There are many kinds of enzymes in organisms, thousands of which have been found at present. Each enzyme catalyzes different chemical reactions to help complete all chemical reactions in cells. (2) Transmission and storage functions. Some protein transport and store very important biochemical substances, such as metal ions, oxygen, glucose and esters. (3) The functions of movement and structure maintenance. Some proteins can enhance the strength of organisms or play a protective role. (4) Defense functions. Some proteins can resist the invasion or damage of foreign substances, such as immunoglobulins, which can prevent bacteria and viruses from invading, bind or surround foreign substances and make them inactive. (5) Adjustment functions. Some proteins can assist in the regulation of cell activity or its physiological function or bond with DNA to regulate the biosynthesis of enzymes and RNA, such as hormones.

Wang et al. [118] found a large amount of miRNA enrichment in *Rehmannia glutinosa* (*R. glutinosa*). By extracting and separating miRNAs from different parts of *R. glutinosa* root, stem and leaf, the miRNAs were identified. It was found that there were 89 conserved miRNAs and 6 new miRNAs in *R. glutinosa* and that these miRNAs play important roles in transcriptional regulation, plant growth and development, and signal transduction. Shahid et al. [119] showed that a large number of new miRNAs were found in the haustorium of *Semen cuscutae* (*S. cuscutae*). Many of these miRNAs were 22 nucleic acids in length. However, this length of plant miRNAs is not common, and thus was related to the production of secondary siRNAs. During parasitism, 22 nucleic acid length miRNAs were identified as dodder targets some mRNAs of *Arabidopsis thaliana* (*A. thaliana*), which leads to the cutting of mRNA, the production of secondary siRNA and the reduction of mRNA content. Further study showed that the growth rate of *S. cuscutae* after parasitization was significantly accelerated after the mutation of two sites encoding target mRNA in *A. thaliana*, indicating that miRNAs targeting host mRNA are beneficial to the parasitization of dodder. The study also found that the same miRNA was also expressed when dodder parasitized tobacco. The target site of dodder miRNA was also predicted in homologous mRNAs from other plants. The miRNAs of *S. cuscutae* can target host mRNA across species to regulate its gene expression, indicating that these miRNAs play a role in the process of *S. cuscutae* parasitism.

Improving the growth and development of medicinal plants is the basis for the accumulation of active ingredients, and the synthesis of the main active substance secondary metabolites is closely related to primary metabolism.

#### The relationship between primary metabolism and secondary metabolism in medicinal plants

**Fig. 2:**
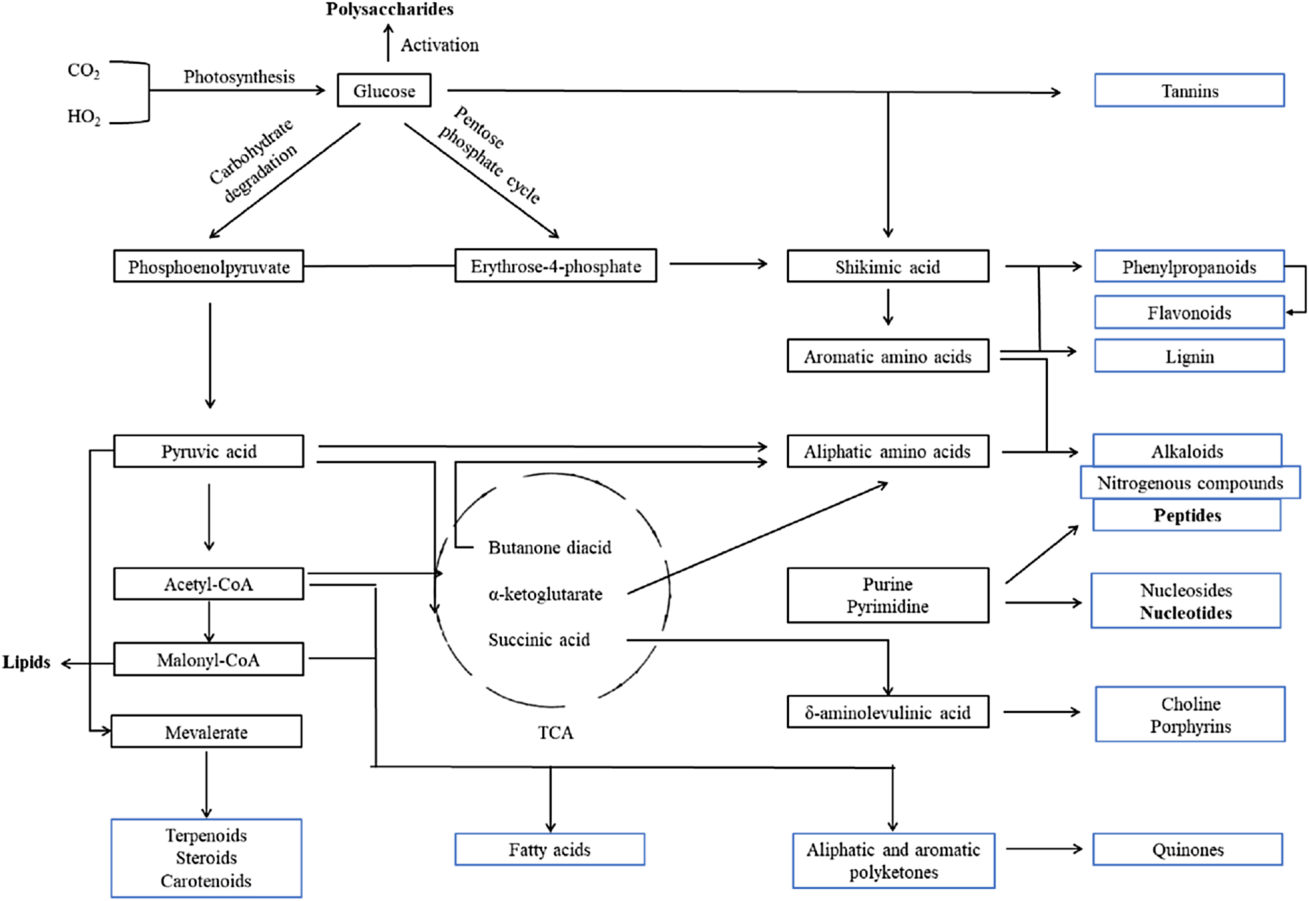
Metabolism and biosynthesis of substances in medicinal plants. The bold content is the macromolecule products of primary metabolism

Primary metabolism is directly related to the growth, development and reproduction of plants, providing energy and intermediate products for the survival, growth, development and reproduction of plants. Green plants and algae hydrate carbon dioxide into sugars through photosynthesis and further produce ATP, NADH, pyruvic acid, phosphoenolpyruvate, 4-monophosphate-erythrose, ribose and other essential substances to maintain the life activities of plant flesh using different mechanisms [120]. Phosphoenolpyruvic acid and 4-phosphate-erythritose can further synthesize shikimic acid (the starting material of the shikimic acid pathway), while pyruvic acid can generate acetyl CoA (the starting material of plant secondary metabolism) after hydrogenation and decarboxylation. These products then enter the citric acid cycle to generate a series of organic acids and malonic acid monoacyl CoA, along with other products, and a series of amino acids (including substrates of nitrogen compounds). These processes are primary metabolic processes. Under certain conditions, some important primary metabolites, such as acetyl coenzyme A, malonyl coenzyme A, shikimic acid and some amino acids, are used as raw materials or precursors (substrates), and different secondary metabolism processes are further carried out, producing phenolic compounds (such as flavonoids), isoprene compounds (such as terpenes) and nitrogen-containing compounds (such as alkaloids), among other products. In this process, saccharides provide raw materials and a framework for the synthesis of intermediates, and proteins function as the catalytic enzymes for the synthesis of substances [121] (Fig. 2).

The synthesis and accumulation of secondary metabolites in medicinal plants are closely related to the original environment of medicinal materials, which is one of the important reasons for the formation of “genuine” medicinal materials [122]. Since the precursor of many secondary metabolic pathways is the product of primary metabolism, environmental abiotic factors can change the contents of secondary metabolites by affecting primary metabolism. Environmental factors, also known as elicitors, refer to those biological or non-biological elicitors that can stimulate the accumulation of secondary metabolites. After elicitor induction, the CHS gene-related defense mechanism was induced and expressed efficiently. The essence of this efficient expression indicates the improvement of transcription activity. Under stress, photosynthesis, the shikimic acid pathway and amino acid metabolism in plants are all affected. However, environmental factors can affect the amount and/or activity of enzymes in the secondary metabolism pathway, thus affecting the synthesis of secondary metabolites. Several studies have shown that pal gene expression can be affected by environmental factors, such as salinity, light, and carbon dioxide concentration, and this enzyme is the key enzyme of the phenylpropane metabolism pathway. Drought stress can change the synthesis of isoflavones and flavonoids by changing the expression of isoflavone biosynthesis genes (such as CHS, IFS, DFR and 4CL). In addition, drought can also affect the isoprene-like biosynthesis pathway. Under UV irradiation, the activities and quantities of PAL, IFS, CHS, F3H and other enzymes are affected, thus altering the synthesis of related secondary metabolites [123]. Several basic hypotheses about the synthesis of plant secondary metabolites all support the view that adversity is the condition of plant secondary metabolism. Although the secondary metabolites changed obviously under stress conditions, we conjecture that macromolecular substances with high contents, complex structures and relative stability in medicinal plants are the key and essence to answer stresses and maintain a relative balance in plants. The secondary metabolites are the product of interactions between macromolecular substances and the environment. These findings are pending further study.

## Future prospects and conclusion

There are many kinds and complex structures of effective components in TCM, and they have synergistic or antagonistic effects on each other. These substances constitute a complex system of multicomponents, multichannels and multitargets of TCM, bringing great difficulties and challenges to the study of the material basis of TCM. In this review, we found that biomacromolecules play a considerable role in the formation of Chinese medicine efficacy, as evidenced by their biological activities related to TCM efficacy (Table 1). A further analysis of the direct and indirect action mechanisms of biomacromolecules was conducted. In most cases, polysaccharides are absorbed in the form of oligosaccharides, which directly or indirectly affect the intestinal flora to influence efficacy, and the strength of this effect is closely related to their structure. Proteins are mostly absorbed in the form of oligopeptides to enact their functions. miRNA can be absorbed through the gastrointestinal tract and can act as a suppressive signal molecule between cells in the target tissue. Of course, we cannot ignore the cooperative relationship between macromolecular and micromolecular materials, which is the essence of the synergy of TCM. Failure to complete these studies can result in a fatal knowledge gap for a TCM. Such polysaccharides, proteins and miRNAs derived from TCM have a wide range of immune regulatory functions with low side-effects. The immunomodulation of polysaccharides and proteins is a multichannel, multilevel, multitarget process. There are a variety of neurotransmitters, hormones and immune substances that together allow the maintenance of immune function in both sensitive and stable equilibrium, just as the ‘yin and yang, qi and xue organ function’ balanced Holism in TCM (Fig. 1). The growth, development and quality formation of medicinal plants are inseparable systematic processes. The biological functions of biomacromolecules are elaborated from their roles in the process of plant growth and development and in the relationship between primary metabolism and secondary metabolism (Fig. 2).

However, current studies on biomacromolecules still have some limitations. (1) There is no controlled comparison standard for biological activity. Especially in the clinical context, these studies are concentrated in China, Japan, Korea and some other oriental countries; experimental data are still lacking from Europe and the USA. Moreover, most research studies on biomacromolecules (especially proteins) have been separated from the basis and truth that most of TCMs work via decoctions and the process of oral administration into the body. (2) Although many research results on the action mechanisms of polysaccharides and proteins have been reported, the exact molecular immunoregulatory mechanisms of polysaccharides, proteins and miRNAs in immune and signaling pathways are still unclear. In addition, there is a shifting approach regarding polysaccharide receptors and signaling pathways in animals and clinical trials. It seems that polysaccharides and proteins are immunomodulators rather than purely immunopotentiators. Polysaccharides and proteins likely do not exert the same actions in all patients; thus, the mechanism of action in each circumstance should be studied. (3) The chemical structures and chain conformations of polysaccharides and proteins play a vital role in their biological activities. Yet, the exact structures, as well as the relationship between bioactivities and the chemical structures, are still not well established, especially for proteins. Moreover, a majority of polysaccharides and proteins in current research use are crude extracts, which are easily affected by other active ingredients of Chinese medicine, making it difficult to determine their exact roles and mechanisms.

Thus, we should strengthen the following aspects. (1) The extraction, separation, purification and detection methods of macromolecular substances should be optimized, improving research into the structure–function relationships and mechanisms of polysaccharides and proteins, especially regarding the higher structure, activity center, and active fragments, which will be helpful to clarify the action mechanism at the molecular level. (2) The research on bioactive biomacromolecules of TCM cannot be separated from the practical applications of TCM. The research process must consider the differences in macromolecular substances in TCM from different sources, such as fresh herbs, processing, and water decoction. Research on bioactive substances should be based on in vivo experiments to more closely model the actual absorption and metabolism properties of the human body. (3) The characteristics of the integration and coordination of TCM should be studied in combination, using active tracers, structure–activity relationships between macromolecules and small molecules, strengthening immune neuroendocrine networks and the development of TCM compounds. Most of the proteins and nucleic acids in TCM macromolecules are thermally unstable, and the effects of these substances in crude drugs and water decoctions are spatiotemporal differences and can be verified. These studies would allow a better understanding of the functional effects of these macromolecules and provide new insight for further development of natural resources. It is time to put thoughts into words and words into action.
